# Indigenous Dengue Fever, Buenos Aires, Argentina

**DOI:** 10.3201/eid1409.080143

**Published:** 2008-09

**Authors:** Marcela Natiello, Viviana Ritacco, María Alejandra Morales, Bettina Deodato, Marisa Picollo, Edith Dinerstein, Delia Enria

**Affiliations:** Hospital “F. J. Muñiz,” Buenos Aires, Argentina (M. Natiello, B. Deodato, M. Picollo); Consejo Nacional de Investigaciones Científicas y Técnicas CONICET, Buenos Aires (V. Ritacco); Hospital Interzonal de Agudos “Evita,” Lanús, Argentina (E. Dinerstein); Instituto Nacional de Enfermedades Virales Humanas "Dr. Julio I. Maiztegui,”, Pergamino, Argentina (M.A. Morales, D. Enria)

**Keywords:** Dengue, Argentina, South America, letter

To the Editor: For 2 decades dengue has increased in the Americas, with epidemic peaks every 3 to 5 years (*1*). The disease has reemerged in 3 South American countries bordering Argentina, namely, Bolivia, Brazil, and Paraguay.

Argentina had remained free from dengue for >80 years before the disease was reintroduced in 1998 (*2*) as a consequence of insufficient mosquito control and importation of cases from disease-epidemic areas. Since then, indigenous dengue circulation has only been reported in the northern provinces of the country, which are close to endemoepidemic countries. However, the principal dengue vector, the *Aedes aegypti* mosquito,*,* has spread southward to latitude 35°S near Buenos Aires (*3*).

We describe what might be the southernmost indigenous case of dengue fever documented in South America; this case occurred in 2007, an epidemic peak year for the disease on this continent (*1*). The patient was a pneumonologist who worked part-time at Muñiz Hospital, a referral infectious diseases treatment center in the Buenos Aires Federal District. She also provided healthcare at an outpatient clinic in Lanus, her town of residence, a suburb 6 km south of the Federal District. Febrile illness started suddenly in February 2007, midsummer season in Argentina. On day 5 of illness, fever was replaced by a short-lived rash and itching followed by asthenia and nausea that persisted for 2 days. The patient had not traveled or been accidentally exposed to patients’ blood during the previous weeks. **She had never been vaccinated against yellow fever.** D**engue fever was only suspected retrospectively.**

Serologic results provided supportive evidence of a recent dengue infection i.e., presence of i**mmunoglobulin M, as determined by** antibody-capture enzyme immunoassay, and **immunoglobulin G seroconversion by 90% plaque reduction neutralization test** on Vero cells (*4*). As shown in the Table, dengue virus serotype **3 was identified, and** a**ntibody results were negative for 3 other flaviviruses.** Thus, this case fulfils Pan American Health Organization criteria for the diagnosis of dengue fever (*5*). Household contacts were seronegative.

**Table:** Serologic findings of an autochthonous case of dengue fever, Buenos Aires, February 2007

Date (days after onset)	MAC-ELISA*	Plaque reduction neutralization test (90%)						
		Saint Louis encephalitis virus	West Nile virus	Yellow fever virus	Dengue 1 virus	Dengue 2 virus	Dengue 3 virus	Dengue 4 virus
2007 Jul 7 (16)	+	<20	<20	<20	80	<20	80	<20
2007 Apr 13 (53)	ND	<20	<20	<20	40	<20	640	<20

*Immunoglobulin M antibody-capture enzyme immunoassay with suckling mouse dengue virus antigen mixture of dengue 1, dengue 2, dengue 3, and dengue 4 serotypes. ND, not determined.

For several years, conditions have been set for dengue virus circulation in Buenos Aires’ urban and suburban areas because of the abundance of mosquitoes and disease in persons recently returning from neighboring countries. Risk for vector transmission is highest in the peripheral quarters of the city and towards late summer (*6*). Besides, Buenos Aires, like other Latin American metropolitan areas, is undergoing demographic changes that convey further risk for mosquito-borne disease transmission, namely, accelerated population growth mainly caused by informal settlements, deficient public health infrastructure and basic services, unregulated immigration from neighboring countries, and increased international mobility especially in or from neighboring countries (*1*).

Only imported dengue cases have been previously documented in Buenos Aires (*2*). **According to official information, all 158 cases confirmed by antibody conversion in Buenos Aires Federal District and Province** **during 2007 were also imported (*7*). Of these,** 50 occurred in the southern suburban district where our patient lives and works. In **the summer of 2007, dengue infection was mainly introduced into the area by Paraguayan natives living in Buenos Aires who had recently visited their homeland. Dengue 3 serotype conversion was demonstrated in most of the cases investigated by plaque reduction neutralization assay, except for a few cases imported from Brazil, in which dengue 1 serotype was detected.**

Most of the patients whose cases were diagnosed in Buenos Aires, including 5 who required hospitalization, were referred to Muñiz Hospital. Built a century ago, Muñiz Hospital comprises a number of independent pavilions surrounded by a spacious garden, where mosquitoes thrive, especially in summer. Thus, vector-borne infection in this case might have occurred either in Muñiz Hospital, in the Federal District, or in the southern city suburb, where the patient lives and works.

Until recently, dengue had not been suspected in patients with a fever living in the Buenos Aires area in the absence of a recent history of travel to an endemoepidemic area. Confirmation of our case was evidence of local circulation of dengue virus. Thereafter, serum testing became recommended in Buenos Aires for acute febrile illness, among other dengue surveillance interventions in the area. More recently, epidemiologic surveillance of febrile illness has been strengthened countrywide upon the recent reporting of yellow fever cases in Argentina (*8*).

No circulation of dengue virus was reported in Buenos Aires during the first 10 epidemiologic weeks of 2008. However, vector control measures should be strengthened to minimize the risk of infective persons triggering an epidemic of dengue or other flavivirus disease.
